# Truth Stranger Than Fiction

**Published:** 1896-01

**Authors:** 


### TRUTH STRANGER THAN FICTION.

A novelist who should say that a nail could be driven into a child's brain and remain there without producing any unpleasant symptoms for thirty-two years, would be thought to exceed even the license of the romancer. And yet this fact was revealed in the dead-house of the Metropolitan Hospital, at a recent autopsy. Among the daily arrivals of patients from the city was a man thirty-two years old, of fair intelligence and apparently well formed, suffering from double pneumonia. In forty-eight hours he was carried to the dead-house, and in accordance to hospital rules, all cases of sudden death, in due time, a post-mortem examination was made. As the skull cap was lifted, a nail was found which had passed through the skull and penetrated, for three-quarters of an inch, into that portion of the brain which is supposed to be the seat of thought. The head of the nail was imbedded in the skull, and covered by the scalp with its full growth of hair, showing that it had passed through the soft portions in babyhood. Inquiry into the previous history of the dead man showed that he was a laborer, had lived all his life in New York, had never suffered until the attack of pneumonia from any special disease, was of usual intelligence, and had never complained of headache, and yet, during all these years, he had carried this nail penetrating into the brain, the rust, when removed, staining the surrounding brain-cells and scaling when touched with a knife from the nail itself. It was one of the strange revelations which the physician meets in the wards and the dead-house of a great hospital, showing how often truth is stranger than fiction.—*New York Medical Times*, September, 1895.

